# Haplotype-resolved genome assembly and genome-wide association study identifies the candidate gene closely related to sugar content and tuber yield in *Solanum tuberosum*

**DOI:** 10.1093/hr/uhaf075

**Published:** 2025-04-10

**Authors:** Lei Gong, Li Zhang, Haiwen Zhang, Fengjie Nie, Zhenning Liu, Xuan Liu, Miaoquan Fang, Wenjing Yang, Yu Zhang, Guohui Zhang, Zhiqian Guo, Hongxia Zhang

**Affiliations:** Guyuan Branch Academy of Ningxia Academy of Agriculture and Forestry Science, 200 Yiwu Road, Guyuan, 756000 Ningxia Hui Nationality Autonomous Region, China; Ningxia Academy of Agriculture and Forestry Science, 590 Huanghe East Road, Yinchuan, 750002 Ningxia Hui Nationality Autonomous Region, China; Peking University Institute of Advanced Agricultural Sciences, 699 Binhu Road, Xiashan District, Weifang, 261325 Shandong Province, China; Ningxia Key Laboratory for Agro-biotechnology, Research Center of Agricultural Biotechnology, Ningxia Academy of Agriculture and Forestry Science, 590 Huanghe East Road, Yinchuan, 750002 Ningxia Hui Nationality Autonomous Region, China; College of Agriculture and Forestry Science, Linyi University, Middle Section of Shuangling Road, Linyi, 276000 Shandong Province, China; Ningxia Key Laboratory for Agro-biotechnology, Research Center of Agricultural Biotechnology, Ningxia Academy of Agriculture and Forestry Science, 590 Huanghe East Road, Yinchuan, 750002 Ningxia Hui Nationality Autonomous Region, China; Huazhi Biotechnology Co. Ltd, 618 Heping Road, Furong District, Changsha, 410016 Hunan, China; Ningxia Key Laboratory for Agro-biotechnology, Research Center of Agricultural Biotechnology, Ningxia Academy of Agriculture and Forestry Science, 590 Huanghe East Road, Yinchuan, 750002 Ningxia Hui Nationality Autonomous Region, China; Ningxia Key Laboratory for Agro-biotechnology, Research Center of Agricultural Biotechnology, Ningxia Academy of Agriculture and Forestry Science, 590 Huanghe East Road, Yinchuan, 750002 Ningxia Hui Nationality Autonomous Region, China; Guyuan Branch Academy of Ningxia Academy of Agriculture and Forestry Science, 200 Yiwu Road, Guyuan, 756000 Ningxia Hui Nationality Autonomous Region, China; Guyuan Branch Academy of Ningxia Academy of Agriculture and Forestry Science, 200 Yiwu Road, Guyuan, 756000 Ningxia Hui Nationality Autonomous Region, China; College of Agriculture and Forestry Science, Linyi University, Middle Section of Shuangling Road, Linyi, 276000 Shandong Province, China

## Abstract

As an important noncereal food crop grown worldwide, the genetic improvement of potato in tuber yield and quality is largely constrained due to the lacking of a high-quality reference genome and understanding of the regulatory mechanism underlying the formation of superior alleles. Here, a chromosome-scale haplotype-resolved genome assembled from an anther-cultured progeny of ‘Ningshu 15’, a tetraploid variety featured by its high starch content and drought resistance was presented. The assembled genome size was 1.653 Gb, with a contig N50 of approximately 1.4 Mb and a scaffold N50 of 61 Mb. The long terminal repeat assembly index score of the two identified haplotypes of ‘Ningshu 15’ was 11.62 and 11.94, respectively. Comparative genomic analysis revealed that positive selection occurred in gene families related to starch, sucrose, fructose and mannose metabolism, and carotenoid biosynthesis. Further genome-wide association study in 141 accessions identified a total number of 53 quantitative trait loci related to fructose, glucose, and sucrose content. Among them, a tonoplast sugar transporter encoding gene, *StTST2*, closely associated with glucose content was identified. Constitutive expression of *StTST2* in potato and Arabidopsis increased the photosynthetic rate, chlorophyll and sugar content, biomass tuber and seed production in transgenic plants. In addition, co-immunoprecipitation assays demonstrated that StTST2 directly interacted with SUT2. Our study provides a high-quality genome assembly and new genetic locus of potato for molecular breeding.

## Introduction

Potatoes (*Solanum tuberosum* L.), ranking as the fourth most widely cultivated crop globally, are pivotal in agriculture due to their nutritional benefits and versatile applications, including food, animal feed, thickening agent, and bioethanol production [1–3]. As the global population is projected to reach 9.8 billion by 2050, there is an imperative demand for the genetic advancements to enhance potato yield and quality.

The rapid progress in molecular breeding for staple crops like rice, maize, and wheat has highlighted the urgency of establishing a high-quality reference genome for potato [4–7]. The tetraploid nature of most cultivated varieties, coupled with high heterozygosity and complex inheritance patterns, presented significant challenges for genomic assembly [2, 3, 8–11]. Despite the assembly of draft genomes for diploid potato lines ‘RH89–039–16’ and ‘DM1–3516R44’ over a decade ago, many gaps still persisted [12]. Advances in single-molecule real-time (SMRT) sequencing have since enabled the construction of improved reference haplotype (RH) and diploid model (DM) genomes, including the gap-free DM8.1, which has elucidated the genomic distribution of agronomically important gene clusters [8, 10]. Recent assemblies of haplotype-resolved genomes for three tetraploid cultivars and pan-genomic analyses of 44 diploid genomes have further enriched the genetic resources available for potato research [13, 14].

Despite these advancements, there remains a critical need to sequence diverse potato varieties to comprehensively understand the genetic determinants of key agricultural traits. Previous genetic studies mainly relied on the linkage analysis in two-parent populations, yielding limited genetic loci with low accuracy due to genomic complexity and population constraints [4, 15, 16]. Hundreds of loci controlling tuber yield and starch content have been mapped across the potato genome, often forming gene clusters ranging from 0.5 to 4 M bp in width [17]. To deepen our understanding on the genetic factors influencing agriculturally relevant traits, an F_1_ tetraploid mapping population derived from ‘Russet’ potatoes has been employed, revealing significant QTLs associated with glucose content and fry color on chromosomes 4, 5, 6, 10, and 11 [15].

**Figure 1:**
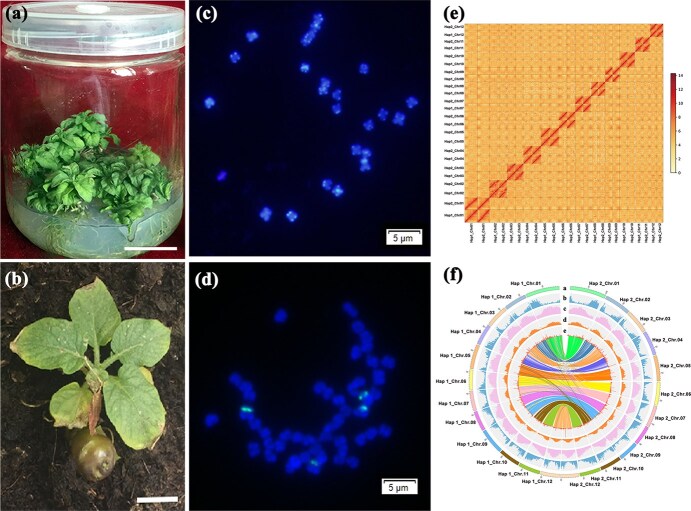
Phenotypes, chromosome analyses and genomic assembly of ‘N8–4’. (a) Anther-cultured heterozygous diploid ‘N8–4’ seedlings from tetraploid ‘N15’. Scale bar = 2 cm. (b) A representative of ‘N8–4’ tubers. Scale bar = 2 cm. (c, d) Fluorescence in situ hybridization (FISH) patterns of ‘N8–4’ and ‘N15’. Chromosomes were stained with 4′-6-diamidino-2-phenylindole (DAPI). FISH signal detection of ‘N8–4’ was conducted with a telomere specific repeat probe (figure c), and FISH signal detection of ‘N15’ was conducted with 5S rDNA repeat sequence probe (figure d). Scale bar = 5 μm. (e) Hi-C interaction heatmap of 12 chromosomes from two haplotypes of the ‘N8–4’ genome. The resolution of the heatmap is 500 kb and dark red dot indicates high contact probability. (f) Genomic features of the two haplotypes. Tracks from outer (a) to inner (e) rings indicate the following: length (Mb) of chromosomes; gene density; TEs or transposons; guanine-cytosine (GC) content; gene expression levels. The transcript level for each gene was estimated by averaging the fragments per kb exon model per million mapped reads (FPKM) from different tissues. For each allelic pair, the longer chromosome was associated with haplotype 1, and the shorter chromosome was associated with haplotype 2. Syntenic relationships between the two haplotypes were connected with lines in the center. All statistics were calculated using 1 MB windows.

Genome-wide association studies (GWAS) were instrumental in identifying genome-phenotype associations across various crops [6, 18]. However, GWAS in potatoes were limited due to the absence of a high-quality autotetraploid reference genome. Schönhals *et al.* identified 89 SNP alleles associated with tuber yield and starch content using RADseq and the 8.3 k SolCAP SNP genotyping array, marking a significant step forward in potato GWAS [17]. Furthermore, a chromosome-scale reference genome for ‘Qingshu 9’ and subsequent GWAS with 141 tetraploid potatoes has unveiled gene correlated with tuber flesh color [14].

In this study, we present a high-quality genome from the anther-cultured plantlets of the potato variety ‘Ningshu15’ (N15), characterized for its high starch content and drought resistance [19]. We also performed GWAS for tuber sugar content in 141 tetraploid accessions and identified the tonoplast sugar transporter (TST) as a regulator of glucose content and yield. Our findings demonstrate that StTST2 interacts with SUT2 to enhance the expression of sugar metabolism genes, offering valuable insights into the genetic improvement of yield.

## Results

### Genomic assembly and annotation

‘N8–4’, a diploid variant, generated from anther cultures of tetraploid ‘N15’ (2*n* = 4X = 48), which characterized with its elite features of high starch content and drought resistance, served as material for genomic sequencing and assembly. Compared with ‘N15’, the growth of ‘N8–4’ was severely restrained, exhibiting extremely dwarfed growth phenotype (Fig. 1a and b; Supplementary Fig. S1a-c). Although no significant change in starch content was observed, plant heights and fresh weights of N8–4 plants were significantly decreased (Supplementary Fig. S1a-c).

Employing a combination of 17-mer depth distribution analysis and flow cytometry, we estimated that the haplotype genome size of ‘N8–4’ was approximately 899.2 Mb, with a heterozygosity rate of 1.77% (Table 1). Given the significant genomic heterozygosity, a high-quality heterozygous diploid potato genome was assembled through merging the data obtained via three different sequencing and assembly strategies. Illumina short fragment sequencing yielded a total of 138.54 Gb of raw reads and PacBio long fragment sequencing yielded a total of 128.76 Gb of raw reads, providing approximately 297-fold genomic coverage (Supplementary Table S1). The draft genome was then assembled using Pacbio long reads, resulting in two haplotypes. Haplotype 1 (828.2 Mb) and haplotype 2 (825.8 Mb) consisted of 364 and 428 scaffolds with N50 lengths of 64.8 and 61.8 Mb, respectively. A total of 151.6 Gb Hi-C reads were generated and used to scaffold the sequences of each group into chromosomes. As a result, 95.95% of the contigs were anchored to the 12 chromosomes of haplotype 1, encompassing a total length of 828.1 M bp. Similarly, 93.12% of the contigs were mapped to the 12 chromosomes of haplotype 2, with a total length of 825.73 M bp. To assess the accuracy of the genome assembly, selected reads from a short-insert library were aligned to the assembled genome using the BWA software. The results indicated that the short reads mapping rate for haplotype 1 and haplotype 2 was 95.07% and 95.06%, respectively.

**Table 1:** Summary of the ‘N8–4’ genomic assembly and annotation.

Genomic assembly index	Total	
Estimated genome size (Mb)	1696.71	
	Haplotype 1	Haplotype 2
Assembly size (Mb)	828.21	825.83
N50 of scaffolds (Mb)	64.77	61.78
N50 of contigs (Mb)	1.47	1.48
Repeat sequences (%)	62.34	55.41
LTRs (%)	51.57	45.56
TE proportion (%) Protein-coding genes	61.11 33 868	53.61 35 280
BUSCO (%)	94.2	93.6
LAI	11.62	11.94

BUSCO: Benchmarking Universal Single-Copy Orthologs; LAI: Long terminal repeat (LTR).

Finally, we employed ALLHiC algorithm for allele-defined genome assembly, which phased the two haplotypes to a final length of 828.21 Mb for haplotype 1 and 825.83 Mb for haplotype (Fig. 1e, Table 1). The final N50 values for contigs and scaffolds were 1.47 and 64.77 Mb for haplotype 1, and 1.48 and 61.78 Mb for haplotype 2 (Table 1). The ‘N8–4’ genomic assembly spanned a total of 1654.04 Mb, with approximately 94.5% (1563.67 Mb) of sequences anchored to chromosomes (Supplementary Table S2). The results of Benchmarking Universal Single-Copy Orthologs (BUSCO) indices of 94.2% and 93.6%, and long terminal repeat (LTR) assembly indices (LAI) of 11.62 and 11.94 validated the high quality of the ‘N8–4’ assembly (Table 1, Supplementary Table S3).

Transposable elements (TEs) integral to eukaryotic genomes accounted for approximately 58% (948.85 Mb) of the assembled genome, with 48% represented by intact LTRs (Table 1; Supplementary Table S2). Based on homology comparisons and multiple RNA-seq datasets, a total of 35 280 and 33 868 high-confidence protein-coding genes were, respectively, predicted for haplotypes 1 and 2 (Table 1; Supplementary Table S4). The predicted genes averaged 1070 bp in length, with an average of 4.7 exons per gene (Supplementary Table S4). These annotated genes were interspersed with a significant number of TEs and other repetitive sequences, which constituted 19% of the genome’s total length. The GC content and gene density across each chromosome were found to be similar. For haplotype 1, approximately 77.2% of the genes had homologs in Swiss-Prot and 97.8% in TrEMBL databases. Additionally, 72.7%, 81.9%, 56.7%, and 75.9% of the genes were annotated in the KEGG, InterPro, GO, and Pfam databases, respectively. For haplotype 2, the corresponding percentages were 76.4% in Swiss-Prot, 97.5% in TrEMBL, and 72.1%, 81.2%, 56.4%, and 75.4% in KEGG, InterPro, GO, and Pfam, respectively (Supplementary Table S6).

### Comparative genomics and synteny analyses

To study the genetic relationships between ‘N8–4’ and other related plant species, we conducted a phylogenomic analysis including *Solanum lycopersicum*, *Capsicum annuum*, *Nicotiana tabacum*, *Solanum tuberosum* (DM v6.1), and *Ipomoea batatas*. These species shared a total of 10 873 orthogroups, with 239 of them being single-copy groups (Supplementary Fig. S2a). Notably, we identified 46 and 91 orthogroups unique to the two ‘N8–4’ haplotypes, and 13 positively selected genes (*P* < 0.01) generated from the process by which the frequency of advantageous alleles increased due to natural selection (Supplementary Fig. S2a and c). Among the 13 positively selected genes, three of them were related to sugar metabolism and enriched in pathways such as starch and sucrose metabolism, fructose and mannose metabolism, and amino sugar and nucleotide sugar metabolism, and one of them was related to flavonoid metabolism and enriched in the carotenoid biosynthesis pathway (Supplementary Fig. S2d and e).

In haplotype 1, expansion and contraction, respectively, occurred in 747 and 892 gene families, whereas in haplotype 2, expansion and contraction, respectively, occurred in 686 and 675 gene families (Supplementary Fig. S2b). According to KEGG enrichment analysis, the expanded gene families in both haplotypes were primarily involved in starch and sucrose metabolism, ribosome, and protein processing in endoplasmic reticulum (*P* < 0.05; Supplementary Fig. S2f and g). In comparison, the contracted gene families were primarily involved in photosynthesis, plant hormone signal transduction, and starch and sucrose metabolism (*P* < 0.05; Supplementary Fig. S2h and i). These findings demonstrate significant changes across gene families related to sugar metabolism, with the above pathways playing important roles in potato tuber yield and quality.

**Figure 2:**
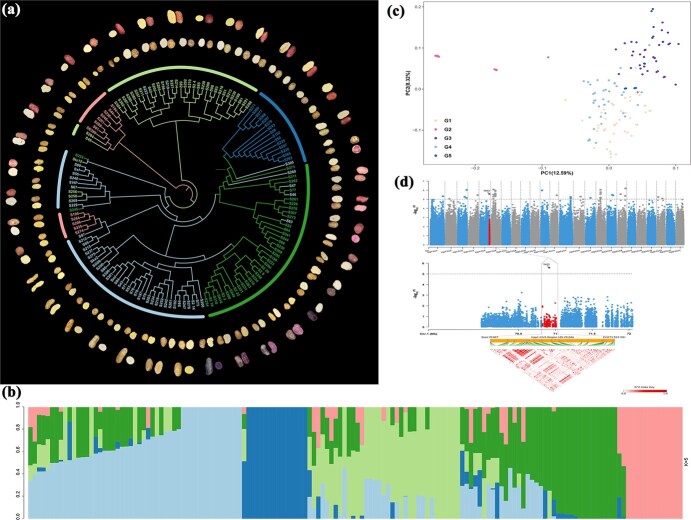
Phenotypes of potato accessions, GWAS of tuber glucose content and identification of *TST2*. (a) Tuber diversity of the 87 potato accessions and phylogenetic tree of the GWAS population. (b) STRUCTURE analysis showing the population of five different subgroups. (c) PCA of 141 accessions using high-quality SNPs from re-sequenced data. Dots in different colors represent different clusters, respectively. (d) Manhattan plots showing the sole significant signal for glucose content on chromosome 5, and Regional Manhattan plot and pairwise LD analysis within the 185 kb interval. Dashed line indicates the significance threshold (−log_10_(*P*) = 5).

The collinearity analysis demonstrated a strong conservation of gene order across both haplotypes in the highly heterozygous autotetraploid ‘N8–4’ (Supplementary Fig. S3a and b). However, the accumulation of structural variations may have contributed to the loss of synteny (Supplementary Fig. S3c). To be more specific, a total number of 7 duplications, 235 translocations, and 86 inversions between haplotype 1 and DM, and 39 duplications, 197 translocations, and 87 inversions between haplotype2 and DM, were observed. In terms of the length of variations, the length of inversions was much larger than other types of rearrangements, with a total size of up to 144.4 Mb. Although most of these inversions were located in the central regions with lower gene density, they still contained nearly 3.6% of all genes (2526 out of 69 148 genes). Collectively, these findings indicate that the new genomic assembly is of high quality and will facilitate the discovery of genes related to important traits.

### GWAS of tuber sugar content and identification of *TST2*

To identify loci associated with tuber glucose, fructose, and sucrose content, whole-genome re-sequencing was conducted on a panel of 141 accessions with diverse genetic backgrounds (Supplementary Table S8). The re-sequenced reads were mapped to the ‘N8–4’ assembly, resulting in a total of 18.4 billion mapped reads with an average depth of 9.9× and an average coverage of 72% for each accession (Supplementary Table S10). After filtering, 17 961 859 single nucleotide polymorphisms (SNPs) were mapped to the assembly and used for subsequent GWAS and population genetics analyses (Supplementary Table S9).

Population genetic analysis was carried out with three complementary approaches, NJ tree, PCA, and genetic structure (Fig. 2a-c). They gave largely consistent results, indicating that distinct genetic differences existed among the five clusters. Cluster 1 contained the largest number (49/141) of accessions, accompanied with the highest average glucose content, whereas cluster 2 contained the smallest number (15/141) of accessions, accompanied with the lowest average glucose content. Clusters 3 and 4 exhibited a mixture of their genetic components, whereas clusters 2 and 5 displayed a relatively simple genetic composition. Generally, accessions with close phylogenetic relationship and similar phenotypic traits were grouped in the same cluster. For example, Qingshu 9 (S239), Tongshu 23 (S249), and Ningshu 15 (S204), which all showed high resistance to drought resistance, were simultaneously grouped in cluster 4 (Fig. 2a-c). The genetic composition of each variety was also revealed with the results of genetic structure analyses. For example, corresponded with their actual genetic backgrounds, Qingshu 9 and Ningshu 15, each of them consisted of two, whereas Tongshu 23 consisted of three, parental lineages (Fig. 2b). This concordance between the genetic structure and known genetic backgrounds substantiated the reliability of the genetic analysis results of the accession population.

To enhance the accuracy of the association analysis, three models were employed for the marker-trait association analysis: general linear model (GLM), mixed linear model (MLM), and fixed and random model circulating probability unification (FarmCPU) (Supplementary Fig. S4). A total of 53 overlapping signals associated with tuber sucrose, fructose, and glucose content were identified (Supplementary Table S11). For both haplotypes, the subgenome-specific loci appeared to be under biased selection and exhibited an asymmetric distribution. Specifically, 32 significant SNPs located on 10 different chromosomes were identified for haplotype 1, and 21 SNPs located on eight different chromosomes were identified for haplotype 2. Notably, chromosome 6 contained the greatest number of SNPs (13/32) for the haplotype 1, while chromosome 4 contained the least number of SNPs (7/21) for haplotype 2 (Supplementary Table S11).

**Figure 3:**
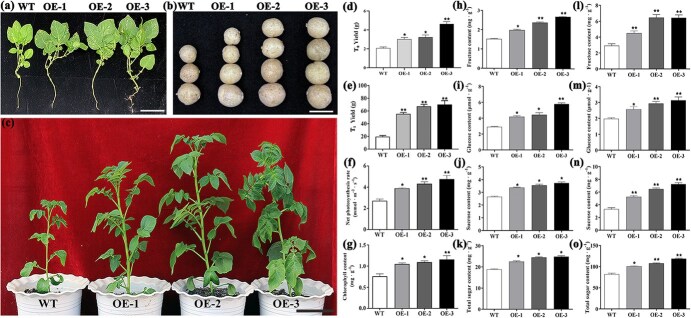
Overexpression of *StTST2* in potato plants. (a) 90-day-old T_0_ transgenic potato plants. Scale bar = 5 cm. (b) Tuber phenotype of wild type (WT) and T_0_ overexpression (OE) plants. Scale bar = 3 cm. (c) 60-day-old T_1_ transgenic potato plants. Scale bar = 15 cm. (d, e) Yield of WT and OE T_0_ and T_1_ plants. (f, g) Net photosynthetic rate and leaf chlorophyll content of WT and OE plants. (h-k) Leaf fructose, glucose, sucrose and total sugar content of WT and OE plants. (l-o) Tuber fructose, glucose, sucrose and total sugar content of WT and OE plants. Bars indicate the standard error (*n* = 3 biological replicates; Student’s *t*-test: ^*^, *P* < 0.05; ^**^, *P* < 0.01).

Among the 53 SNPs, four of them were associated with fructose content, 11 of them were associated with sucrose content, and 38 of them were associated with glucose content (Fig. 2d; Supplementary Table S11). These SNPs accounted for approximately −6.6% to 3.6% of the observed phenotypic variation and exhibited linkage disequilibrium (LD) (Supplementary Table S11). It is worth noting that ten of the SNPs negatively affected agronomic traits, which were evenly distributed on different chromosomes in both haplotypes. Notably, haplotype 2 harbored five of these SNPs and exhibited stronger negative effects (Supplementary Table S11). These results demonstrate that potato tuber sugar content is controlled by multiple genes, and that some of the advantageous alleles are regularly distributed across specific chromosomal regions.

Several genes with known functions were found to be located within corresponding QTLs, including *CONSTANS-LIKE 10* (*COL10*) in the flowering time QTL and *NRT2.4* in the nitrate transporter QTL (Supplementary Table S12). We further analyzed the sole peak with the highest value (5.6) on chromosome 5 (SNP_70918516_), and identified 26 candidate genes within the 185 kb interval (Fig. 2d; Supplementary Table S12). Although none of the identified genes have been reported to be associated with sugar metabolism, one candidate gene, *TST*, encoding a monosaccharide-sensing protein, was located approximately 622 kb away from our mapping interval. And this gene has been previously reported to be essential for chlorophyll biosynthesis and tuber quality [20–22]. To validate the reliability of the gene downstream of the strongest association signal, we conducted two complementary analyses. First, we searched for all annotated genes within a 622 kb interval downstream of the strongest association signal and identified 86 genes, none of them were involved in sugar metabolism regulation (Supplementary Table S13). Second, we performed expression analysis of *TST2* in leaves from six representative cultivated varieties and observed significant differences in its relative expression levels (Supplementary Fig. S5). Combining these analyses, we preliminarily characterized *TST2* as the candidate locus associated with sugar content regulation.

### *StTST2* alters tuber sugar content and increases tuber yield

To further confirm the role of *TST2* in sugar metabolism and other agronomic traits, we overexpressed *TST2* in cultivar ‘Atlantic’. Compared to wild-type (WT) plants, *StTST2* overexpressing (OE) plants exhibited significant improvement in biomass accumulation and tuber yield (Fig. 3a and c). Both T_0_ and T_1_ OE plants produced larger tubers than WT plants, resulting in higher tuber yield (Fig. 3b, d, e). Furthermore, *StTST2*-OE plants accumulated significantly more sugar (fructose, glucose, sucrose, and total sugars) in leaves than did WT plants (Fig. 3h-k). In addition, *StTST2*-OE plants exhibited higher photosynthetic efficiency and chlorophyll content than did WT plants (Fig. 3f and g).

**Figure 4:**
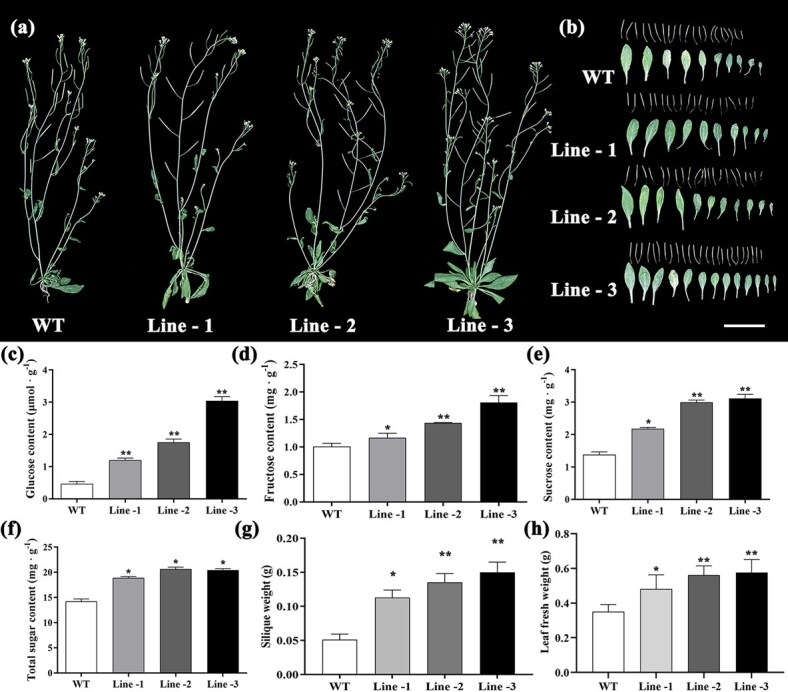
Ectopic expression of *StTST2* promoted the growth of transgenic Arabidopsis. (a) Six-week-old transgenic Arabidopsis plantlets. Scale bar = 5 cm. (b) Leaf and seed phenotypes of wild type (WT) and transgenic Lines. (c-f) Leaf fructose, glucose, sucrose and total sugar content of WT and different lines. (g, h) Comparison of seed weight and fresh leaf weight of WT and different lines. Bars indicate the standard error (*n* = 3 biological replicates; Student’s *t*-test: ^*^, *P* < 0.01; ^**^, *P* < 0.01).

We further heterologously expressed *StTST2* in *Arabidopsis thaliana.* Similar results were also observed in transgenic Arabidopsis lines. Overall, the fresh leaf weight and seed weight of the three independent lines was significantly higher than those of WT plants (Fig. 4a, b, g, h). Furthermore, sugar content (fructose, glucose, sucrose, and total sugars) increased at least one-fold in transgenic plants compared to that in WT plants (Fig. 4c-f). Therefore, *StTST2* not only increased photosynthetic efficiency and sugar content, but also enhanced plant growth and biomass production.

### TST2 interacts with SUT2 to increase the expression of sugar metabolism-related genes

**Figure 5:**
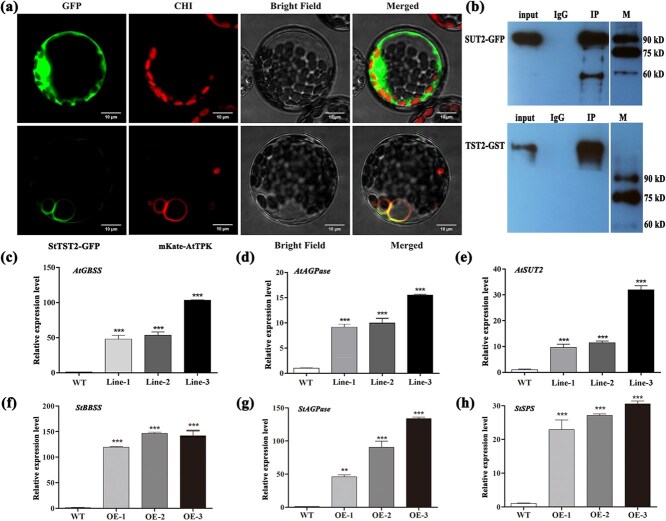
Subcellular localization of StTST2 and expression of sugar metabolism-related genes in wild-type (WT) and *StTST2*-overexpressing plants. (a) Subcellular localization of StTST2. The *35S::GFP* and *35S::StTST2-GFP* vectors were transiently expressed in Arabidopsis protoplasts. The results were visualized under a confocal microscopy 16 h after transformation. CHI, chloroplast autofluorescence; marker protein, mKate-AtTPK, homomeric vacuolar channel protein. Scale bar = 10 μm. (b) Co-Immunoprecipitation (Co-IP) assays to confirm the interaction between StTST2 and SUT2. The GFP-tagged *SUT2* was immunoprecipitated by GST-tagged *StTST2*, as detected by Western blotting. The presence of *SUT2* and *StTST2* was detected by immunoblotting using GFP and GST antibodies. Lane 1, input; Lane 2, IgG; Lane 3, IP; GFP, 23 kD; SUT2-GFP, 93 kD; StTST2-GST, 107 kD. (c-e) Relative expression of *GBSS*, *AGPase* and *SUT2* in WT and transgenic Arabidopsis lines. (f-h) Relative expression levels of sugar metabolism-related genes (*GBSS*, *AGPase*, and *SPS*) in WT and transgenic Arabidopsis lines. *Ef1a* and *actin* were used as reference genes. Bars indicate the standard deviation (*n* = 3 technical replicates; Student’s *t*-test: ^*^, *P* < 0.05. ^**^, *P* < 0.01.^***^, *P* < 0.001).

The StTST2 protein, which was predicted to be involved in monosaccharide sensing and transport, was found to be localized to the vacuolar membrane (Fig. 5a). Furthermore, StTST2 was predicted to interact with SUT2, and their physical interaction was confirmed through Co-Immunoprecipitation (Co-IP) assays in transiently-transformed tobacco (Fig. 5b).

To further elucidate the involvement of *StTST2* in sugar metabolism, we examined the expression of genes regulating sugar metabolism. Heterologous expression of *StTST2* in Arabidopsis plants resulted in increased expression of genes encoding ADP-glucose pyrophosphorylase (AGPase), granule-bound starch synthase II (GBSSII) and sucrose transporter (SUT2) (Fig. 5C-E). Moreover, the expression levels of *AGPase*, *GBSSII*, and sucrose phosphate synthase (*SPS/SuPS*) were significantly increased in OE potato plants (Fig. 5f-h). These findings suggest that *StTST2* could be involved in sugar transport and storage to regulate sugar metabolism and cellular partitioning.

## Discussion

Potatoes are a globally significant crop, ranking at the fourth position in cultivation right after wheat, rice, and maize [3]. However, the complexities of tetrasomic inheritance, highly heterozygous genomes and the historical lack of a high-quality reference genome have impeded molecular genetic improvement in potato breeding [9, 10, 13]. We presented a chromosomal-level genome, and conducted GWAS analysis on 141 tetraploid potato accessions and identified 53 loci associated with glucose and sucrose content. Among these, we performed preliminary functional validation one locus, *TST2*.

### Advancements in potato genomic research and distinctive features of the ‘N8–4’ reference genome

Since the initial partial assembly of the first reference genome for a monoploid potato (DM1–3516 R44) in the year of 2011, significant progress has been made in potato genomics [12]. The high-resolution, haplotype-level assembly of the heterozygous diploid potato genome (RH89–039–16), which integrated whole genome sequencing (WGS), 10x Genomics linked-read sequencing, Oxford Nanopore Technology (ONT) and Hi-C, has landed a notable milestone [8]. The use of high-fidelity (HiFi) reads from Circular Consensus Sequencing was shown to be particularly transformative for assembling reference genomes of highly heterozygous polyploid crops, leading to several chromosome-level genomes that have been invaluable for genomic and genetic research in potato [9, 14, 23]. Recently, a phased potato pangenome graph of 60 haplotypes was developed and enhanced heterozygosity as well as deleterious structural variants in cultivated diploids were observed [24]. This research offers a comprehensive insight into the haplotype diversity of a clonal crop, laying the theoretical groundwork for the reimagining of potato.

The progression of sequencing technologies has highlighted the pressing need for supplementary reference genomes of tetraploid potatoes to fully encompass the genetic diversity inherent within the potato varieties. Ningshu 15, a tetraploid cultivar known for its high starch content and drought resistance, was anther-cultured to generate N8–4 [19, 25]. The N8–4 genome offers a valuable comparative genomic tool against previous monoploid (like DM) and diploid (like RH) potato varieties. This new assembly not only expands the reference genome database for popular commercial strains, including Cooperation-88, ‘Atlantic’, and Qingshu 9, but is also essential for exploring how ploidy levels, structural variation relate to key potato traits like yield and resistance [2, 3, 14, 26].

The ‘N8–4’ genome exhibited a higher heterozygosity rate of 1.77%, surpassing ‘Otava’ (1.53%) and ‘Atlantic’ (1.76%) [3, 9]. To resolve the complex genome at the haplotype level, we sequenced it using Illumina short fragment sequencing, PacBio long fragment sequencing and Hi-C technology. The ‘N8–4’ haplotypes with genome sizes of 825.8 and 828.2 Mb were considerably larger than the genome (741.6 Mb) of ‘DM’ V6.1 [23]. Despite this, the scaffold N50 values of the ‘N8–4’ haplotypes (61.8 and 64.8 Mb) were superior to those of Cooperation-88 (59.9 Mb) and Qingshu 9 (46.24 Mb), indicating a contiguous assembly [2, 14]. Hi-C contact matrix further confirmed the quality of our phased assembly (Fig. 1e).

### Genetic structure and diversity of GWAS population

The number of re-sequenced materials, the genetic structure of the population and the depth of sequencing are the key factors significantly influencing the accuracy for identifying trait-associated loci of GWAS. Based on recent researches involving 108–370 tetraploid potatoes collected globally, GWAS was conducted to investigate the population structure and genetic diversity of these germplasm resources [7, 14, 27]. The classification of the GWAS population into three to five distinct subgroups suggests a complex genetic architecture (Fig. 2a-c). Each subgroup demonstrates low genetic diversity, indicating that although extensive allelic diversity exists across the entire population, the genetic variation within each subgroup is limited.

Consistent with population structures from previous studies, our analysis revealed that the 141 accessions could be categorized into five distinct clusters, underscoring the genetic diversity’s consistency (Fig. 2a and b). These clusters showed intertwined genetic components, suggesting complex genetic interactions and the potential for hybrid vigor. Variations in glucose content across clusters indicated that genetic variation affected agronomic traits. Notably, varieties from the same breeding unit were often grouped together, reflecting shared breeding histories and the accumulation of specific alleles through selection (Fig. 2a and b). These findings underscore the genetic structure of GWAS population in elucidating the genetic basis of agronomic traits in tetraploid potatoes.

### Identification and insights into sugar-related SNPs

Tuber yield and sugar content are critical for the agronomic performance of potatoes, with sucrose being the key carbon compound for the synthesis of transitory starch in leaves and its storage in tubers. Despite its significance, the genomic organization and regulatory mechanisms of carbohydrate metabolism-related genes in potatoes remain underexplored, highlighting a need for genomic studies to unravel the genetic underpinnings of these traits [1, 28, 29].

Previous studies have identified four significant QTLs related to tuber glucose content in an F_1_ tetraploid mapping population [15]. Through genotyping array analysis, 89 SNP alleles were found to have synergistic effects on tuber yield, starch content and starch yield [17]. Our GWAS identified 53 loci associated with sucrose, fructose, and glucose content (Fig. 2d; Supplementary Table S11). Notably, 20 of the identified SNPs were located on chromosomes 4 and 6, aligning with previous reports [1, 15, 17]. Additionally, novel QTLs controlling glucose and sucrose contents were identified on chromosomes 1, 2, 7, 11, and 12, with SNPs having more severe adverse effects predominantly found in haplotype 2 (Supplementary Table S11). Most of the identified SNPs had minor effects and were distributed in intergenic or intronic regions, highlighting the polygenic nature of sugar content and the challenges in pinpointing functional genes. These observations are consistent with previous study that it was difficult to detect minor effect QTLs for sugar content or yield due to their discontinuous distribution and linkage to deleterious mutations using conventional genetic methods or small populations [29]. Therefore, the ongoing efforts to identify novel genes controlling these important traits in potato are crucial for facilitating molecular breeding aimed at improving yield. The identification of these SNPs and QTLs underscores the importance of high-accuracy reference genomes for identifying functional genes.

### TST2 promotes sugar accumulation and yield formation

The genetic factors influencing tuber yield and starch content remain largely unknown [30]. In this study, a TST was identified and proved to have a crucial role in sugar accumulation and biomass in both potato and Arabidopsis. Previously known as tonoplast monosaccharide transporter (TMT), this transporter was first identified in rice and Arabidopsis [20, 31]. In these plants, TST/TMT was found to increase the vacuolar glucose content of mesophyll cells and to increase seed yield. In addition, it has been demonstrated that *StTST1* was crucial for sugar accumulation and starch metabolism regulation following postharvest cold storage [21]. We found that *StTST2* significantly increased glucose, fructose, sucrose, and total sugar content in both the leaves and tubers of transgenic potato plants (Fig. 3h-k). Similar results were also reported in previous studies on sweet sorghum, melon, watermelon, apple, and tomato [32–35]. In addition, overexpression of *StTST2* resulted in improved tuber yield and biomass accumulation (Figs 3a and b and 4a and b). These results are complementary to the observation in *StTST3.1*-silenced potato plants, which exhibited reduced sucrose, hexose and chlorophyll contents in leaves [22]. Collectively, our findings indicate that *TST* is essential for the partitioning of sugars within cells, particularly for the loading of glucose and sucrose into the vacuole. TST-mediated intracellular sugar allocation promotes sugar accumulation in leaves, which in turn may serve to regulate photosynthesis and promote the generation of more assimilates.

Previous research suggests that TSTs regulate sugar accumulation and redistribution by mediating the transport of glucose and sucrose into vacuoles. For example, the CBL2-CIPK6-TST2 module was required for glucose transport in cotton (*Gossypium hirsutum*) [36]. MdERDL6 modulated vacuolar sugar accumulation by modulating the function of MdTST1/2 in apple (*Malus domestica*) [32]. We predicted the downstream interacting proteins of TST2 and found that it interacted with SUT2 to promote its expression (Fig. 5b and e). We speculated that the physical interaction between TST2 and SUT2 might be pivotal for managing intracellular sugar partitioning and for yield regulation. This interaction may directly enhance the expression of *AGPase*, *GBSSII*, *SPS*, and *SUT2*, thereby promote the transport of sucrose from leaves to tubers (Fig. 5c-h). *SUT2* functioned as a sucrose sensor, releaser and transporter, and played a crucial role in phloem loading and the export of sucrose from leaves to stems [37–40]. Moreover, SUT1/SUC2, SUT2/SUC3, and SUT4 interacted with one another to create oligomeric complexes that could potentially regulate sucrose transport [41, 42]. Expression of SUT2 was upregulated by exogenous glucose and sucrose [43]. Taken together, *TST2*, *SUT2*, and *SUT* oligomeric complexes could induce a signal transduction cascade affecting the expression of certain downstream genes, and form a molecular signaling feedback loop with photoassimilates (glucose and sucrose), ultimately affecting photosynthesis and sugar transport [44, 45]. Our results offer experimental support for this hypothesis and highlight the importance of the TST2-SUT2 module in transporting sucrose from the vacuole to the cytosol, and in regulating cellular sugar allocation and yield determination.

**Figure 6:**
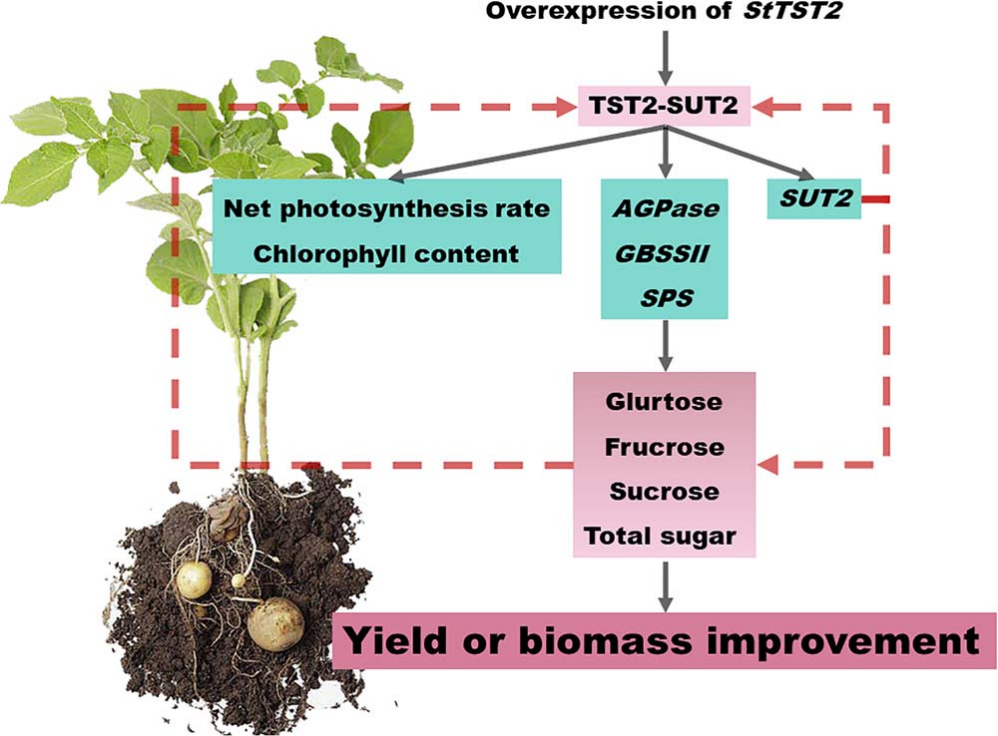
A proposed *StTST2*-mediated model regulating cellular sugar partitioning and tuber yield in potato. StTST2 physically interacts with SUT2 and enhances the expression of genes involved in sugar metabolism, either directly or indirectly, thereby fostering sugar biosynthesis.

In summary, we provided detailed sequencing data and valuable gene resource to support the improvement of potato for enhanced yield and quality. First, we constructed a chromosome-level genome of diploid material, which will be a valuable resource for studies on intra-genomic variation and allele-specific gene expression. Second, 53 novel QTLs associated with sugar content, including *TST2*, were identified via GWAS in 141 tetraploid accessions. Third, we characterized the function of *StTST2* in improving yield and proposed a molecular regulatory model. Moreover, the interaction of StTST2 with SUT2 boosts the expression of genes crucial for the metabolism of sucrose. The upregulated expression of both *StTST2* and *SUT2*, along with the increased sugar content, forms a feedback regulatory loop promoting the source-to-sink transport of sugars, ultimately enhancing yield (Fig. 6).

## Materials and methods

### Plant materials

Sequence materials were derived from the tetraploid potato cultivar ‘Ningshu15’ and its diploid progeny was obtained through anther culture [19, 25]. The karyotypes of ‘Ningshu15’ and its diploid progeny were evaluated through flow cytometry [46]. Leaves from the same plants were sampled for DNA extraction, genomic sequencing and assembly. Fresh potato leaves, flowers, roots, stems and tubers were sampled for RNA extraction, transcriptomic sequencing and gene annotation. In addition, leaf samples of 141 tetraploid potato accessions with diverse genetic backgrounds were collected and used for genomic sequencing and GWAS, which included modern, old, reported cultivars, CIP accessions and breeding lines (Supplementary Table S7). All the materials for resequencing were grown in Xiji County (N105.864897, E35.828638), Ningxia Hui Autonomous Region, in 2020 and 2021.

### Sugar content analysis

Sucrose, glucose and fructose contents were used for association analysis. To determine the sugar content, leaves of each sample in the flowering period were collected and determined on xMark spectrophotometer (BIO-RAD) according to the manufacturer’s manual (D799392, D799404, D799790, D799408, Sangon Biotech Co., Ltd.).

### Genomic sequencing

Genomic DNA was extracted and sequenced using second- and third-generation sequencing platforms with default settings at Novogene Co., Ltd (Beijing, China). High-quality DNA was fragmented and adapters were ligated to the fragments. A paired-end library, with a 300–500 bp insert size, was constructed using an Illumina DNA Prep kit and sequenced using at Illumina HiSeq X platform, according to the manufacturer’s instructions (Illumina Inc, CA, USA).

For third-generation long-fragment sequencing, genomic DNA was fragmented and end-repaired, and adapters were ligated to the fragments. A 10–20 kb DNA library was prepared using the SMRT cell express template Prep Kit 2.0 (Pacific Biosciences, CA, USA). Target fragment size was selected using a Blue Pippin system (Sage Science, MA, USA). DNA library was sequenced at the PacBio Sequel platform with P6/C4 chemistry (Pacific Biosciences, CA, USA).

### Hi-C library preparation and sequencing

Fresh, young potato leaves were sampled and fixed with formaldehyde. Cross-linked DNA samples were digested with restriction enzyme Hind III. Oligonucleotide terminals were labeled with biotin, and ligated proximity DNA fragments were purified and sheared to approximately 350 bp in size. Sequencing libraries were constructed using NEB next ultra-enzymes and Illumina-compatible adapters. After terminal repair, PCR amplification and purification of DNA fragments, DNA library quality was evaluated using Qubit 2.0 and Agilent 2100, and sequenced using an Illumina Hiseq PE150 system (Illumina Inc, San Diego, CA, USA).

### Genomic assembly

After quality control, PacBio read errors were corrected. Next, the pre-assembled reads were used for *de novo* assembly using Falcon v0.732 [47] and Canu v1.5 [48]. Haplotypes were assembled using FALCON-unzip, with the following parameters: max_n_open_files = 1000, unzip_concurrent_jobs = 1000, quiver_concurrent_jobs = 1000, unzip_blasr_concurrent_jobs = 1000, unzip_phasing_concurrent_jobs = 1000. Homologous contigs were further optimized and corrected based on self-alignment and depth. The well-assembled Canu and WTGDB results were merged using Quickmerge. The merged genome was corrected using Quiver and Pilon v1.22 [49].

To improve the accuracy of the genomic sequence, Illumina NGS reads were mapped to the assembled genome using BWA v0.7.10 [50]. Genomic sequence completeness was assessed using the Benchmarking Universal Single-Copy Orthologs (BUSCO) v4.0.5 [51] and Core Eukaryotic Genes Mapping Approach (CEGMA). Continuity was assessed using LTR LAI [52].

The obtained Hi-C reads were mapped to the draft genome using BWA v0.7.10, with default parameters. Duplicate and unmatched data were removed using SAMTOOLS [53]. The scaffolds were clustered to chromosomes using the ALLHIC package, with the following parameters: —NonInformativeRatio 0 —minREs 50 —MaxLinkDensity 3 —filter yes —enz DpnII [54].

### Transcriptomic sequencing

High-quality total RNA was extracted using a plant total RNA isolation kit (Sangon Biotech Co., China). After quality control, RNA samples were subjected to mRNA enrichment with dT probes and fragmented, and the first and second cDNA strands were synthesized. Sequencing adapters were ligated to the double-stranded DNA fragments and amplified via PCR. The constructed library was then sequenced on an Illumina Nextseq 2000 platform (Illumina Inc, San Diego, CA, USA).

### Genomic annotation

The *de novo* repeat sequence library was constructed using LTR_FINDER, RepeatScout, RepeatModeler and Piler [55, 56]. Predicted results were merged using the Repbase homologous repeat sequence database [57]. Genomic repeat sequences were annotated using Repeatmasker. Tandem repeats were analyzed using TRF [58]. Gene structure was predicted using Augustus, GlimmerHMM, SNAP, Geneid and Genscan [59, 60]. Blast and genewise homology prediction were used to align the genomic sequences to the protein database for *Arabidopsis thaliana*, pepper (*Capsicum annuum*), tobacco (*Nicotiana tabacum*), rice (*Oryza sativa*), tomato (*Solanum lycopersicum*), potato (*Solanum tuberosum*), and sweet potato (*Ipomoea batatas*). Transcriptomic sequencing data and homologous species cDNA data were aligned using Blat [61]. Predicted gene models were aligned to proteins in SwissProt (Bairoch and Apweiler, 2000), TrEMBL, the NCBI non-redundant protein database (NR), Pfam (V27.0) and InterPro (v32.0) using Blat (identity >30%) [62]. Predicted genes were also subjected to GO classification and KEGG pathway analysis [63, 64]. Non-coding RNAs were annotated using tRNAscan-SE, RNAmmer, and INFERNAL [65, 66].

### Gene family identification and phylogenetic analysis

Identification of gene families was carried out using OrthoMCL [67]. Single-copy orthologs were aligned in MUSCLE and concatenated into a supergene alignment [68]. RaxML (version 7.2.3) was used to construct a phylogenetic tree utilizing the maximum likelihood (ML) method and GTRGAMMA model [69]. Divergence times were calculated using the PAML package MCMCTREE, with the following parameters: burn-in = 10 000, sample-number = 100 000, sample-frequency = 2 [70]. Calibration points for the divergence analysis were obtained from the TimeTree database [71]. The expansion and contraction of gene families were evaluated using CAFÉ (Version 1.6) [72]. The branch-site model of the codeml tool in PAML was used to test for positive selection. In MCScan X, a syntenic block was defined as collinearity of more than five genes [73].

### Genomic re-sequencing and GWAS

After filtering out adapter sequences and low-quality reads, the clean reads of 141 potato accessions were mapped to our assembled reference genome using BWA, with the following parameters: mem-t 4-k 32-M. SNPs were identified and filtered using SAMtools and Plink [74]. An integrity threshold of >0.9 and a minor allele frequency (MAF) of >0.01 were used to filter highly-consistent SNPs. Principal component analysis (PCA) was carried out using GCTA and EIGENSOFT. A phylogenetic tree was constructed using the neighbor-joining (NJ) algorithm in Treebest. LD was analyzed using PopLDdecay. Population structure was evaluated using admixture. GWAS based on GLM, MLM, and FarmCPU were conducted in GEMMA with default settings at Huazhi Biotechnology Co. Ltd (Changsha, China).

### Generation of *StTST2* transgenic plants

Total RNA was extracted from leaves of potato cultivar ‘Atlantic’ using a Plant Total RNA Isolation kit (Vazyme, Nanjing, China). Total cDNA was reverse transcribed using a HiScript III 1st Strand cDNA Synthesis kit (Vazyme, Nanjing, China), and the *TST2* gene was amplified using PCR primers (Supplementary Table S6). The full-length coding sequence of *TST2* gene fragment (MT454112) was purified and ligated into the pCE2-TA/Blunt-Zero vector. Expression vector pCAMBIA1302-35S::*StTST2* was constructed and transformed into potato cultivar ‘Atlantic’ and Arabidopsis using *Agrobacterium* ‘GV3101’-mediated transformation. Positive plants were screened with kanamycin, hygromycin, and quantitative real-time PCR (Supplementary Fig. S6). Transgenic plants were confirmed by PCR amplification, and seeds were harvested for phenotyping.

### Phenotyping of transgenic plants

The T_1_ generation of *StTST2*-transformed potato was transplanted into greenhouse. Ninety days after transplanting, chlorophyll content in leaves and sugar content in tubers were measured using the detection kits purchased from Sangon Biotech Co., Ltd (Shanghai, China). Chlorophyll content in leaves and sugar content in seeds of the T_3_ generation of *StTST2*-transformed Arabidopsis were measured using the same detection kit.

### Quantitative real-time PCR

Total RNA of transgenic potato and Arabidopsis leaves was extracted and reverse transcribed to cDNA according to the method described above. Quantitative real-time PCR (qRT-PCR) was performed in a 20 μl reaction volume using specific primers and TB GreenTM Premix Ex TaqTM II (Tli RNaseH Plus) (Takara Bio Inc. China). The *Ef1a* and *actin* genes were used as internal references for qRT-PCR (Supplementary Table S7). Three biological replicates and three technical replicates were conducted using the BioRad CFX96 Real-Time PCR instrument (BioRad Laboratories, CA, USA). PCR amplification parameters were as follows: activation at 50°C for 2 min, pre-denaturation at 95°C for 2 min, denaturation at 95°C for 15 s, and annealing at 60°C for 1 min for 40 cycles. Relative gene expression level was calculated using the 2^−ΔΔCt^ method. Primer sequences were shown in Supplementary Table S7.

### Subcellular localization of StTST2 and co-immunoprecipitation assay

The 35S::*StTST2* expression vector, with green fluorescent protein (GFP) tags, was constructed for subcellular localization. The coding region of *StTST2* was fused to the N-terminal of GFP under the control of the CaM 35S promoter. The subcellular localization of the GFP tag was monitored in Arabidopsis protoplasts using confocal microscopy 16 hours after polyethylene glycol (PEG)-mediated transformation as previously described [75].

Candidate proteins interacting with StTST2 were predicted using STRING (https://cn.string-db.org/). The physical association between StTST2 and SUT2 was verified via Co-IP assay. Briefly, cDNA sequences of *StTST2* and *SUT2* were amplified and introduced into the pCAMBIA1301-GST and pCAMBIA1301-GFP vectors and co-expressed in *Nicotiana benthamiana*. Protein extracts were tagged with either GFP antibody or GST antibody, and were detected via western blotting as described by Ma *et al.* [76].

## Supplementary Material

Web_Material_uhaf075

## Data Availability

All the data is available at http://bigd.big.ac.cn/ with the BioSample number of SAMC1075593 for the potato genome assembly of N8–4, the accession number of CRA021150 for HIC sequencing data and the accession number of CRA009721 for the whole genome resequencing data of the 141 varieties.
