# Supplementary figures and images for: Haplotype-resolved genome assembly and genome-wide association study identifies the candidate gene closely related to sugar content and tuber yield in Solanum tuberosum
Source: Hortic Res. 2025 Apr 10;12(6):uhaf075. doi: 10.1093/hr/uhaf075 (PMC12038253; doi:10.1093/hr/uhaf075)

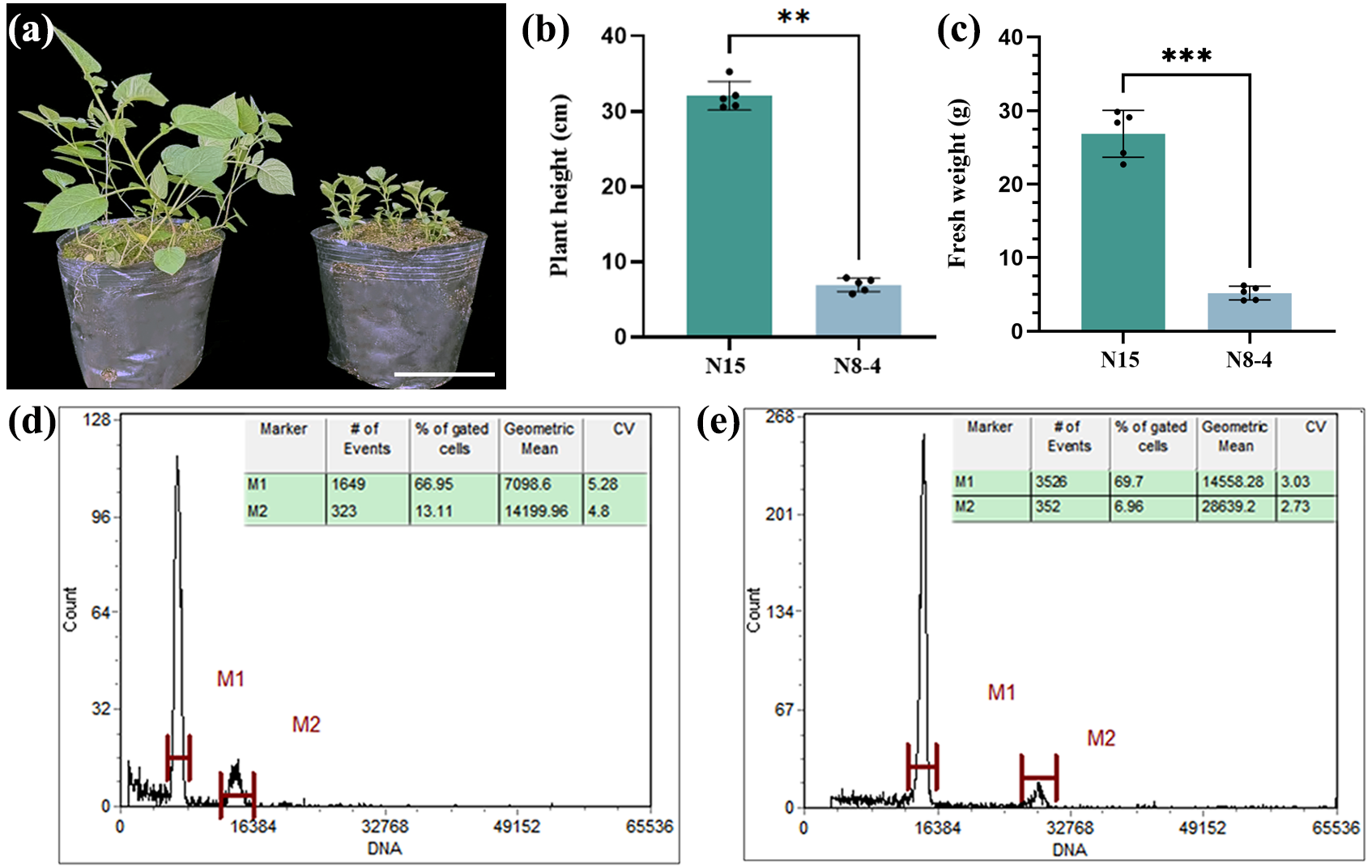

Supplement: Web_Material_uhaf075 [file web_material_uhaf075.zip › Supplementary Fig. S1.tif]

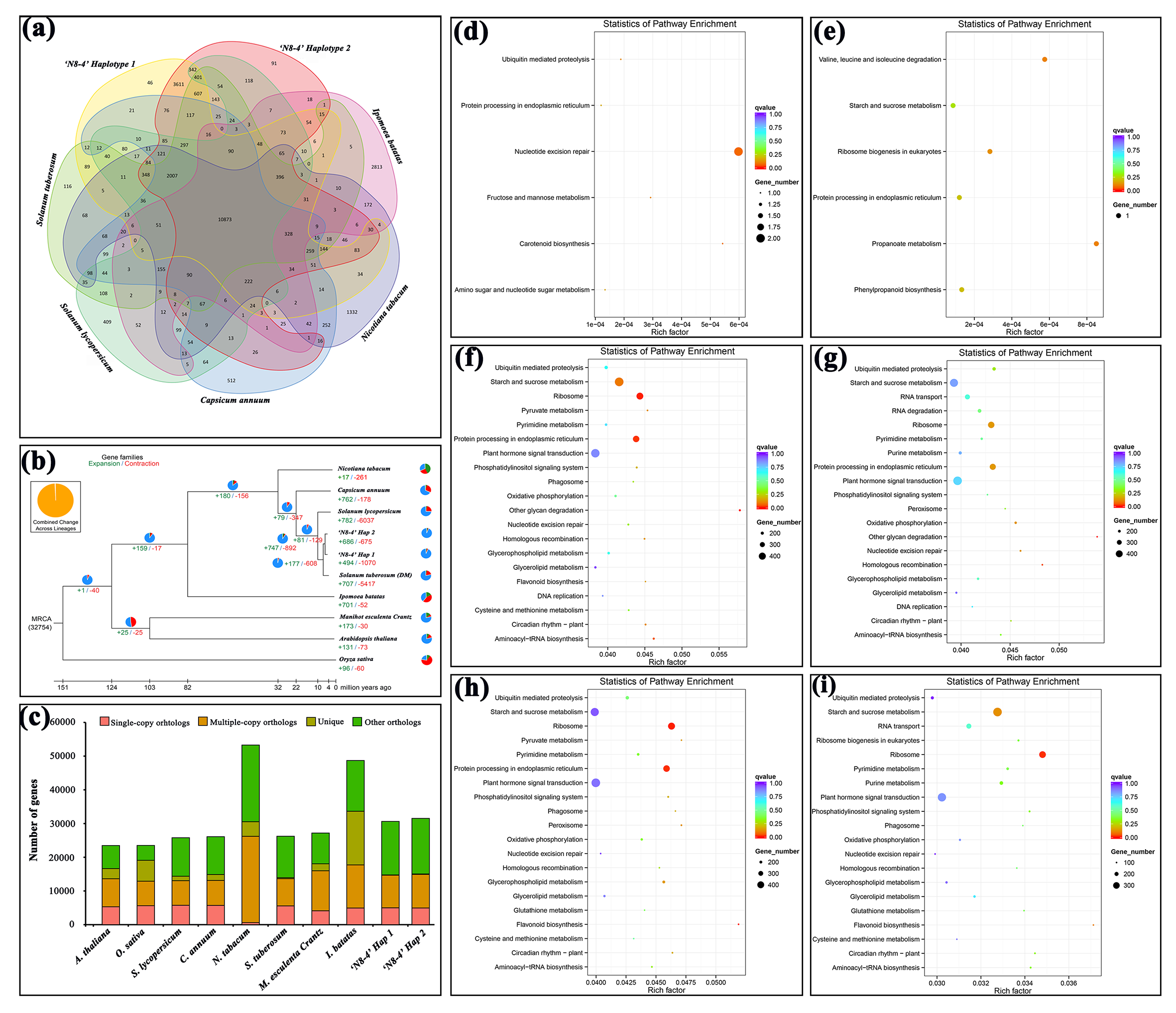

Supplement: Web_Material_uhaf075 [file web_material_uhaf075.zip › Supplementary Fig. S2.tif]

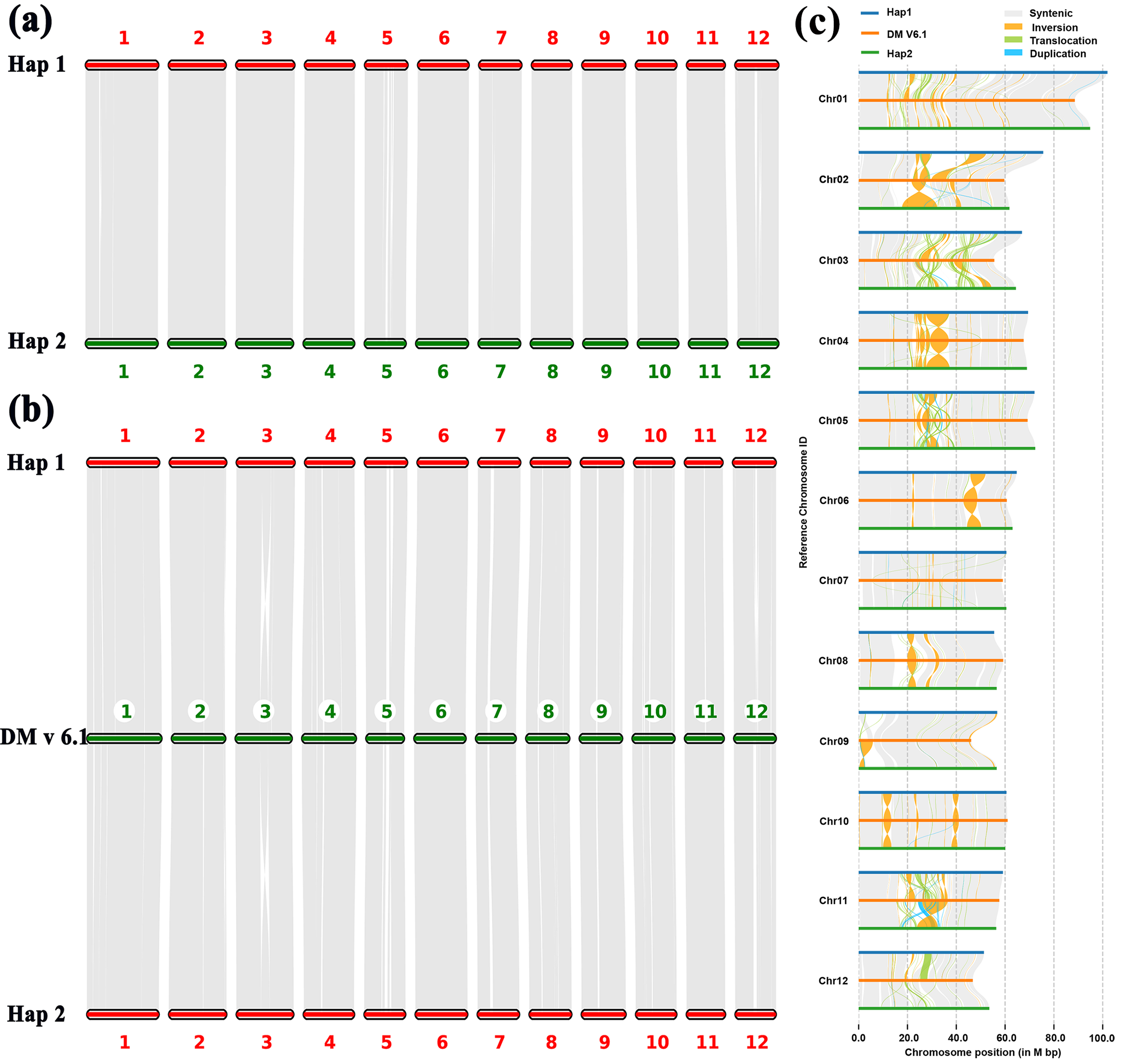

Supplement: Web_Material_uhaf075 [file web_material_uhaf075.zip › Supplementary Fig. S3.tif]

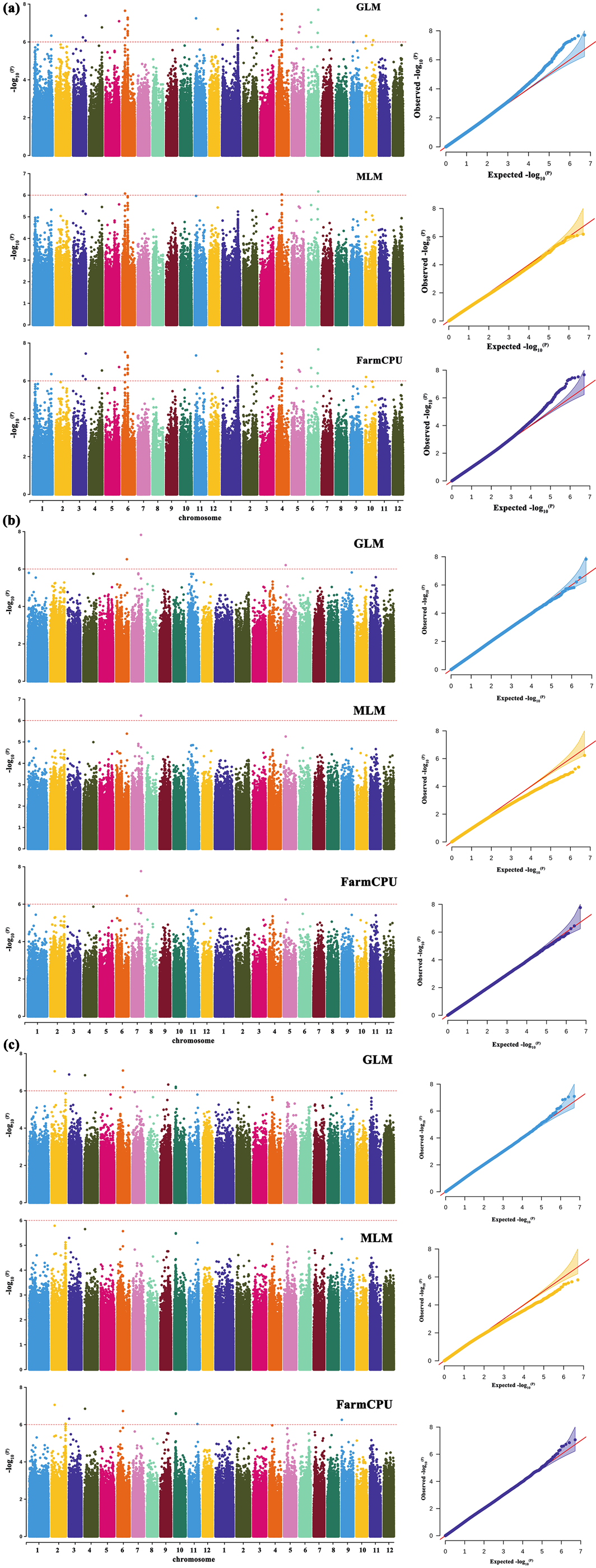

Supplement: Web_Material_uhaf075 [file web_material_uhaf075.zip › Supplementary Fig. S4.tif]

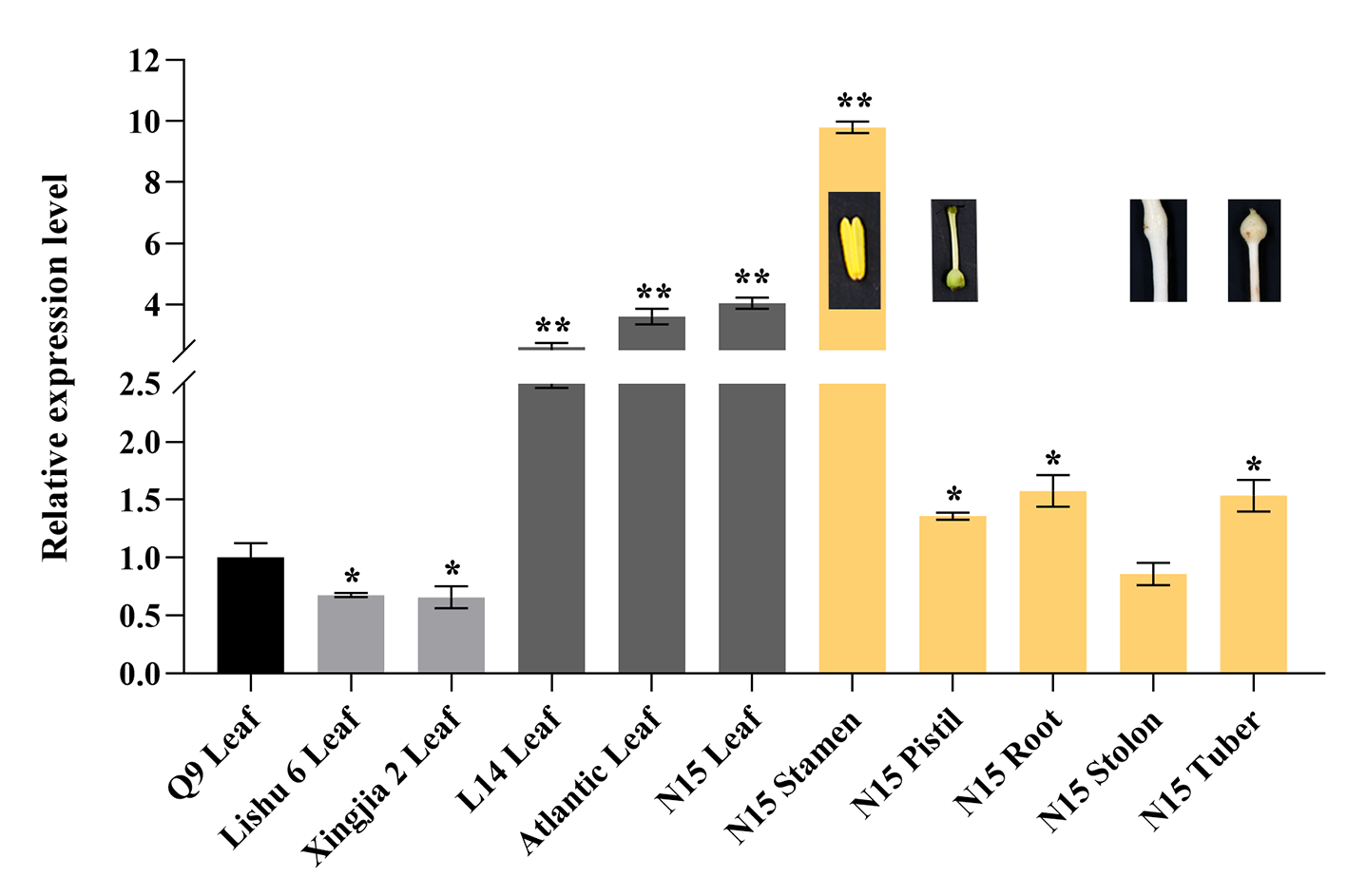

Supplement: Web_Material_uhaf075 [file web_material_uhaf075.zip › Supplementary Fig. S5.tif]

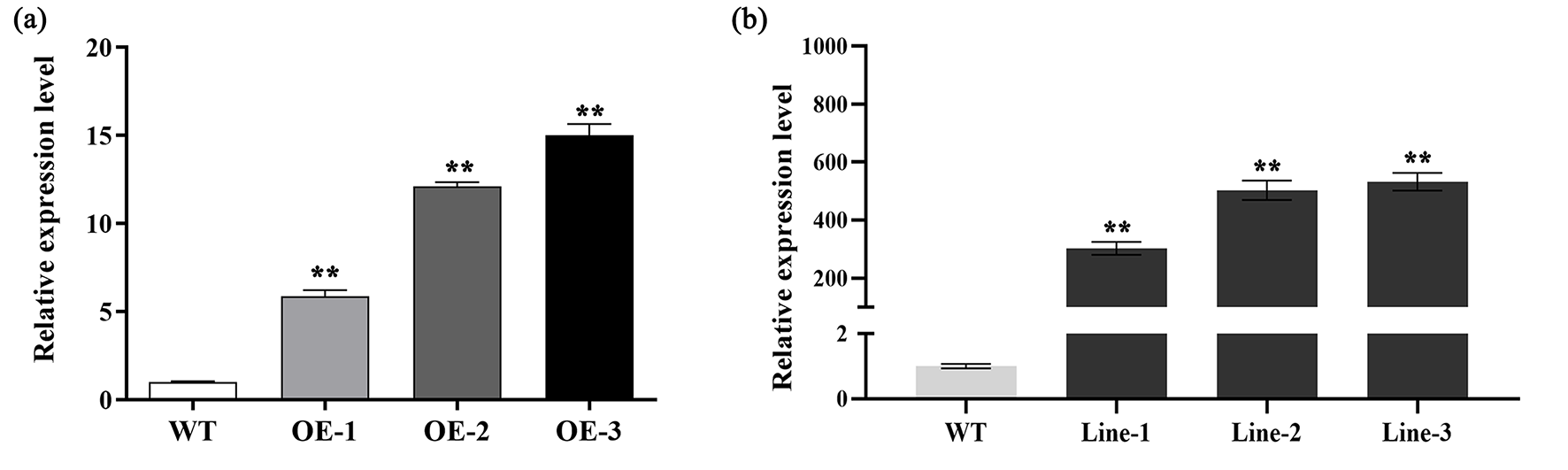

Supplement: Web_Material_uhaf075 [file web_material_uhaf075.zip › Supplementary Fig. S6.tif]

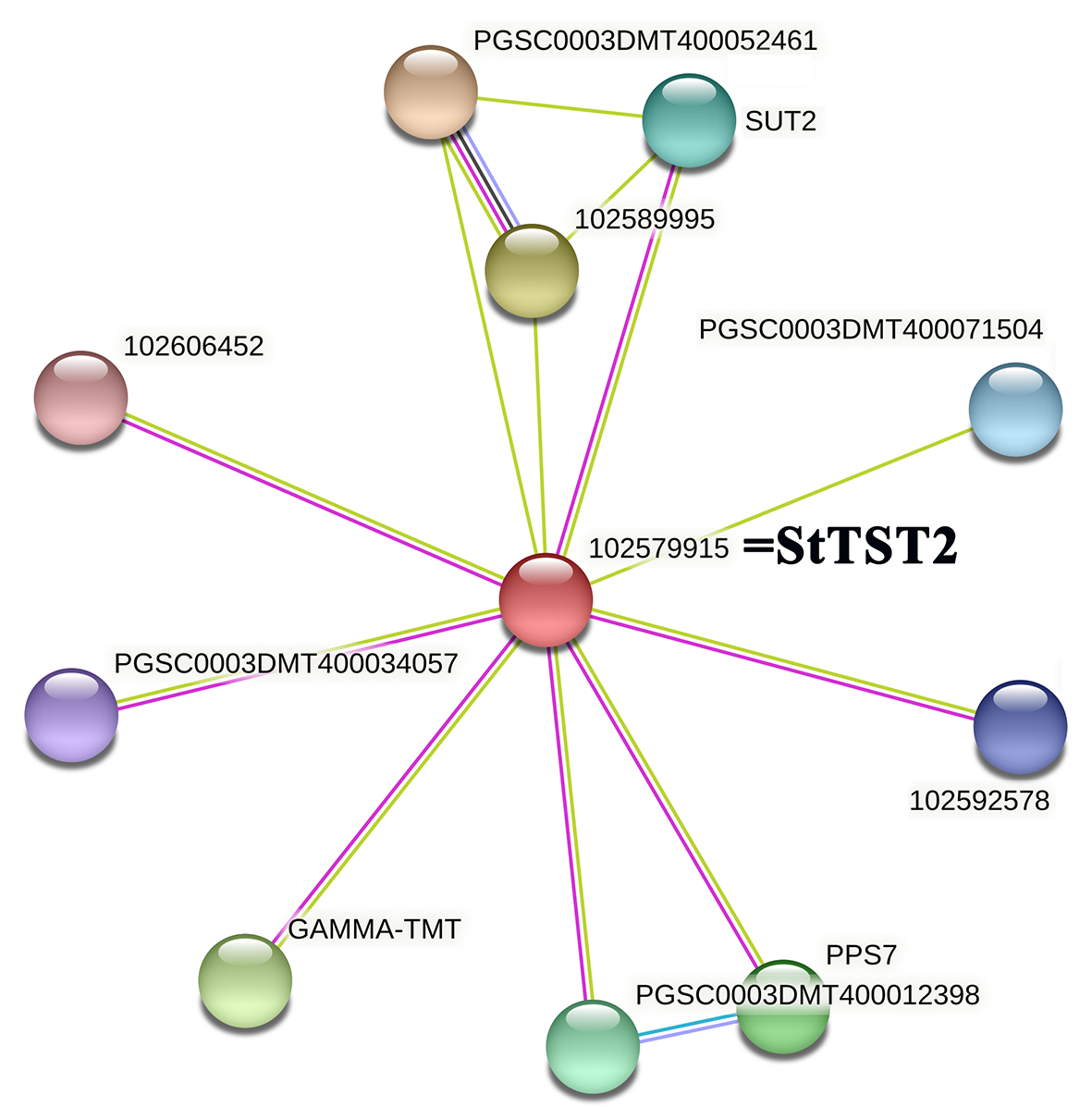

Supplement: Web_Material_uhaf075 [file web_material_uhaf075.zip › Supplementary Fig. S7.tif]
