# Supplementary material for: Haplotype-resolved genome assembly and genome-wide association study identifies the candidate gene closely related to sugar content and tuber yield in Solanum tuberosum
Source: Hortic Res. 2025 Apr 10;12(6):uhaf075. doi: 10.1093/hr/uhaf075 (PMC12038253; doi:10.1093/hr/uhaf075)
Supplement: Web_Material_uhaf075 [file web_material_uhaf075.zip › Supplementary figures-new.doc]

**Title:**

Haplotype-resolved genome assembly and genome-wide association study identifies the candidate gene closely related to sugar content and tuber yield in *Solanum tuberosum*

**Running title:**

*StTST2*, sugar content and tuber yield in potato

**Authors:**

Lei Gong^1, *^, Li Zhang^2^, Haiwen Zhang^3^, Fengjie Nie^4^, Zhenning Liu^5^, Xuan Liu^4^, Miaoquan Fang^6^, Wenjing Yang^4^, Yu Zhang^4^, Guohui Zhang^1^, Zhiqian Guo^1^ and Hongxia Zhang^5, *^

***Corresponding authors:**

Hongxia ZHANG (hxzhang@sibs.ac.cn)

Phone: 86-0535-6664662; Fax: 86-0535-6664660l

**Fig. S1. Phenotype comparison and genome size estimation of ‘N8-4’ and ‘N15’.**

a: Phenotypic state of the tissue culture seedlings after being transplanted into nutrient pots and grown for 8 weeks. Bar = 10 cm.

b: Comparison of plant height and fresh weight after growing in nutrient pots for 8 weeks. The plant height and fresh weight of N15 is three times that of N8-4, significantly higher than the latter (*P*<0.01).

c and d, estimation of nuclear DNA amount (genome size) in ‘N8-4’ (figure a, diploid) and ‘N15’ (figure b, tetraploid) using flow cytometry. The histogram of relative DNA content was obtained after flow cytometric analysis of propidium iodide-stained nuclei of ‘N8-4’ and ‘N15’, which were isolated, stained and analyzed simultaneously. Three randomly selected plants are analyzed.

**Fig. S2.** **Phylogenetic relationship and comparative genomic analyses.**

a: Common and unique gene family’s analysis of the two haplotypes of ‘N8-4’ and the other 8 plant species.

b: Expansions and contractions of gene families. The colors in green and red indicate the expanded and contracted gene families, respectively.

c: The distribution of single-copy, multiple-copy, unique and unclustered genes among the two haplotypes of ‘N8-4’ and the other 8 plant species.

d, e: Positive selection genes of the two haplotypes and their involvement KEGG

f, g: KEGG enrichment of top 20 different gene pathways of the expanded gene families in both haplotypes

h, i: KEGG enrichment of top 20 different gene pathways of the contracted gene families in both haplotypes

**Fig. S3.** **Collinearity and structural variation analysis at chromosome level.** a, collinearity patterns between the two haplotypes of ‘N8-4’. b, Typical macro-collinearity patterns between genomic regions among two haplotypes and DM v6.1. c, Structural variations at the chromosomal level among the two haplotypes of the N8-4 assembly and the DM v6.1 reference genome are demonstrated.

**Fig. S4.** **Manhattan and QQ plots for the association analysis under three models.** a, glucose content. b, fructose content. c, sucrose content.

**Fig. S5. Relative expression level of *StTST2* in different organs of the 6 varieties.** Relative expression level of *StTST2* in leaves of 6 varieties and in the stamen, pistil, root, stolon and tuber of ‘Ningshu 15’.

 **Fig. S6.** **Relative expression level of *StTST2* in selected transgenic potato and Arabidopsis plants.** a, Comparison of *StTST2* relative expression level of leaves from OE and wild-type plants in potato. b, Comparison of *StTST2* relative expression level of leaves from ectopic expression and vector plants in Arabidopsis. Three randomly selected plants are analyzed. Bars indicate the standard error (n = 3 biological replicates; Student’s *t*-test: *, *P* < 0.05; **, *P* < 0.01).

**Fig. S7.** **Predicted protein-protein interaction network of StTST2 via STRING software.** Specifically, purple edge shows known interaction that was determined experimentally. Yellow edge represents the textmining interaction. Blue edge represents for predicted interaction of gene co-occurrence. Black edge shows the co-expression.
